# Early retinal volume changes after switching to brolucizumab in refractory neovascular age-related macular degeneration

**DOI:** 10.3389/fopht.2026.1872499

**Published:** 2026-07-15

**Authors:** Takaaki Matsuki, Kunihiko Akiyama, Ken Watanabe, Toru Noda, Mariko Sasaki

**Affiliations:** 1 Department of Ophthalmology, NHO Tokyo Medical Center, Tokyo, Japan; 2 Division of Vision Research, National Institute of Sensory Organs, NHO Tokyo Medical Center, Tokyo, Japan; 3 Department of Ophthalmology, Keio University of school of Medicine, Tokyo, Japan

**Keywords:** anti-vascular endothelial growth factor therapy, brolucizumab, intraocular inflammation, neovascular age related macular degeneration, switch therapy

## Abstract

**Background:**

This retrospective observational study primarily focused on the evaluation of early anatomical changes and intraocular inflammation (IOI) following the switch to intravitreal injection of brolucizumab (IVBr) in patients with neovascular age-related macular degeneration (nAMD) who were refractory to aflibercept or ranibizumab and unable to extend injection interval beyond 10 weeks while resolving retinal fluids.

**Results:**

Data from 23 patients were analyzed. Switch to brolucizumab resulted in rapid decreases in central foveal retinal thickness (CRT) (247 (130) to 227 (94)) μm, P < 0.001), pigment epithelial detachment volume (0.18 (0.47) to 0.11 (0.19) mm³, P = 0.04) and central choroidal thickness (159 (118) to 168 (78) μm, P = 0.018), although decrease in central retinal volume (CRV) (9.80 (1.40) to 9.70 (1.30) mm³, P = 0.111) was not significant.

Three eyes exhibited IOI, observed on post-injection day 2 after the second IVBr. Two eyes exhibited only mild anterior chamber inflammation, while one eye exhibited vasculitis obliterans. While CRT decreased, CRV increased in all three eyes.

As a *post-hoc* analysis, CRV in eyes without IOI exhibited significant decrease (9.90 (1.45) to 9.70 (1.20) mm³, P = 0.002).

**Conclusion:**

Switch to brolucizumab therapy resulted in early anatomical changes. An increase in CRV despite improvement in central foveal parameters may represent a potential warning sign of brolucizumab-associated IOI and warrants further prospective validation.

## Introduction

Age-related macular degeneration (AMD) is a leading cause of blindness worldwide (1). Anti-vascular endothelial growth factor (VEGF) agents have shown effectiveness in treating neovascular AMD (nAMD) (2, 3), although some patients exhibit resistance, requiring reinjection of anti-VEGF agents within two months (4). The requirement for frequent reinjections imposes a substantial burden on both the healthcare system and patients. Brolucizumab, an anti-VEGF agent introduced in 2019, was engineered to highly penetrate neovascularization due to its low molecular weight and high molarity (5, 6). The HAWK and HARRIER trials demonstrated brolucizumab’s non-inferiority to aflibercept in treatment-naïve nAMD, as well as its capacity to extend injection intervals (7, 8). Furthermore, studies have indicated its efficacy in switch therapy for nAMD patients from aflibercept (9–13). However, intraocular inflammation (IOI) remains a significant concern with brolucizumab, as retinal vasculitis and retinal vascular occlusion can potentially result in irreversible vision loss (14). Prompt detection of retinal vasculitis is crucial to prevent such adverse events.

Previous studies have primarily focused on retinal thickness and qualitative assessment of retinal fluids. However, few have examined retinal volume changes, considering that neovascularization and its exudation occur in three dimensions (15). This study aims to evaluate the efficacy of switching to brolucizumab in patients with nAMD who were recalcitrant to aflibercept or ranibizumab therapy, utilizing central foveal parameters in three dimensions. As a preliminary analysis, central foveal parameters to investigate early signs of IOI was explored.

## Methods

This retrospective observational study was conducted at National Hospital Organization Tokyo Medical Center and was approved by the facility’s Ethics Committee (approval number: R22-052). The study adhered to the tenets of the Declaration of Helsinki. All patient information was de-identified and patient consent was not required. Patient data will not be shared with third parties.

### Patients

We examined patients with nAMD, aged over 50 years, who were switched to brolucizumab 6mg(0.05mL)from aflibercept 2mg(0.05mL) or ranibizumab 0.5mg(0.05mL) between 01/10/2020 and 29/02/2024. Patients were excluded if they presented with serous PED without evidence of neovascularization as determined by fluorescence angiography (FA), indocyanine green angiography (ICGA) and/or optical coherence angiography (OCTA), or if the fundus was poorly visible due to severe opaque media.

The T&E protocol is the standard injection regimen at our institution; however, decisions regarding the extension or shortening of dosing intervals are made immediately after the previous injection rather than just before the next one. Patients do not visit the clinic immediately before the next injection but rather immediately after (within a week) to assess intraocular inflammation. The dosing interval between the last aflibercept or ranibizumab injection and the first intravitreal injection of brolucizumab (IVBr) was maintained within ± 2 weeks of the previous dosing interval (4 to 10 weeks). The interval between the first and second IVBr was also kept within this range (4 to 10 weeks).

### Clinical assessment

The diagnosis and subtypes of nAMD were determined by three retinal specialists (TM, KW, KA) using color fundus photography, OCT, OCTA, FA and ICGA. Patients were classified into three subtypes of macular neovascularization (MNV) based on the Macula Society classification: type 1, type 2, and type 3 (16). Type 1 MNV was further subclassified as polypoidal choroidal vasculopathy (PCV) or other subtype, depending on the presence of polypoidal lesion identified by ICGA.

Pachychoroid-driven MNV (PNV) was determined according to the criteria of Kuranami A et al, which include the absence of drusen except for pachydrusen, the presence of choroidal vascular hyperpermeability identified by ICGA, retinal pigment abnormalities outside MNV lesions, pachyvessels under MNV lesions and/or a history of central serous chorioretinopathy (CSC) (17).

Diagnosis of IOI was made based on the expert opinion from Baumal et al. (18) Briefly, IOI was assessed by meticulous observation of both the anterior and posterior segments using a slit lamp microscope at each visit, and the grading of anterior chamber cells and vitreous cells/haze were based on the published criteria (19, 20). OCT was also used to detect vitreous inflammation as reported by Keane et al. (21) When clinical evidence of posterior segment involvement was evident, FA and ICGA were performed to evaluate retinal vasculitis and vascular occlusion.

OCT parameters and best-corrected visual acuity (BCVA) were assessed at two time points: immediately after the last intravitreal injection before switching to brolucizumab (pre-IVBr visit) and immediately after the second brolucizumab injection (post-IVBr visit). OCT imaging was performed using the ZEISS CIRRUS 5000 HD-OCT (Carl Zeiss Meditec Inc., Dublin, CA) with a 6 x 6 mm mac occlusion ular cube scan and a 5-line raster scan centered on the fovea.

The following OCT parameters were measured or evaluated: central foveal retinal thickness (CRT), defined as the average thickness of the central subfield within a 1 mm diameter circle of the ETDRS (Early Treatment Diabetic Retinopathy Study) grid; central retinal volume (CRV), defined as the total retinal volume bounded by the internal limiting membrane to retinal pigment epithelium within a 6 mm diameter circle centered on the fovea; pigment epithelial detachment volume (PEDV), defined as the volume of retinal pigment epithelial detachment (PED), measured within a 5 mm diameter circle centered on the fovea; and CCT, defined as the thickness between Bruch’s membrane and the sclera, measured vertically at the fovea. Additionally, fluid assessments were made for IRF, located within the retina; SRF, located between the retina and the retinal pigment epithelium; and SPEF, located between the retinal pigment epithelium and Bruch’s membrane. OCT parameters were measured automatically using the built-in software, except for CCT and fluid assessment, which were measured manually with the built-in caliper. The presence of IRF, SRF, and SPEF was assessed qualitatively by identifying dark spots in each retinal compartment using the 5-line raster scan. Measurements were excluded from further analysis if the tracing of retinal layers by the built-in software was inaccurate. BCVA was assessed using Landolt charts and recorded as decimal values. These values were then converted to Log MAR (logarithm of the minimum angle of resolution) units for statistical analysis. As a *post hoc* analysis, CRV in cases without IOI (defined as eCRV) was assessed.

### Statistical analyses

Continuous variables are presented as mean ± standard deviation for age and median (IQR (interquartile range)) for other parameters. Comparisons between pre- and post-IVBr visits were made using Wilcoxon signed-rank test. The percentage of fluid-free eyes was compared between pre- and post-IVBr visits using the McNemar’s test. Statistical significance was defined as p < 0.05. Statistical analyses were performed using commercial software (SPSS Statistics 31; SMART ANALYTICS or Microsoft^®^ Excel^®^ 2016; Microsoft Corporation).

## Results

Fifty-two eyes of 52 patients with nAMD were switched to brolucizumab therapy. Of those, 29 eyes were excluded for the following reasons: deviation from the dosing interval specified in the methods (14 eyes), switch from drugs other than aflibercept or ranibizumab (6 eyes), non-vascularized serous pigment epithelial detachment (PED) (5 eyes), treatment interval exceeding 10 weeks before switch (3 eyes), and poor visibility of the fundus due to opaque media (1 eye). The remaining 23 eyes of 23 patients (14 males and 9 females) were included in the analyses.

The mean age of participants was 79.5 ± 7.6 years. Among the nAMD types, 19 eyes (83%) exhibited type 1 MNV, including 5 eyes (26%) with PCV; 4 eyes (17%) exhibited type 2 MNV; and no eyes exhibited type 3 MNV. Eight eyes with type 1 MNV (including 3 with PCV) were diagnosed with PNV.

The median assessment day after both the previous injection and the second brolucizumab injection was post-injection day 2.0 (IQR, 1.0).

Visual acuity did not change significantly between the pre- and post-IVBr visits (0.30 (0.57) to 0.52 (0.54), P = 0.92) (**Table 1**).

**Table 1:** Mean of parameters at the baseline and after switching to IVBr.

All 23 eyes	N	Baseline	After switching	Difference *	P value
Log MAR unit	23	0.30 (0.57)	0.52 (0.54)	0.00 (0.25)	0.92
CRT (μm)	18	247 (130)	227 (94)	-16 (21)	< 0.001
CRV (mm^3^)	18	9.80 (1.40)	9.70 (1.30)	-0.20 (0.45)	0.111
eCRV (mm^3^)	15	9.90 (1.45)	9.70 (1.20)	-0.20 (0.30)	0.002
PEDV (mm^3^)	17	0.18 (0.47)	0.11 (0.19)	-0.05 (0.16)	0.04
CCT (μm)	23	159 (118)	168 (78)	-11 (30)	0.018
PNV 8 eyes	N	Baseline	After switching	Difference *	P value
Log MAR unit	8	0.26 (0.41)	0.41 (0.49)	-0.12 (0.33)	0.574
CRT (μm)	8	247 (62)	226 (59)	-20 (28)	0.012
CRV (mm^3^)	8	9.85 (0.98)	9.65 (1.05)	-0.10 (0.52)	0.553
eCRV (mm^3^)	6	9.85 (0.88)	9.65 (0.78)	-0.30 (0.50)	0.078
PEDV (mm^3^)	7	0.12 (0.30)	0.07 (0.16)	-0.02 (0.12)	0.017
CCT (μm)	8	172 (132)	157 (122)	-13 (23)	0.05
Non-PNV 15eyes	N	Baseline	After switching	Difference *	P value
Log MAR unit	15	0.30 (0.56)	0.52 (0.54)	0.00 (0.16)	0.824
CRT (μm)	10	273 (167)	255 (131)	-16 (10)	0.025
CRV (mm^3^)	10	9.80 (1.70)	9.70 (1.30)	-0.20 (0.25)	0.102
eCRV (mm^3^)	9	9.90 (1.70)	9.70 (1.50)	-0.20 (0.20)	0.012
PEDV (mm^3^)	10	0.24 (0.41)	0.17 (0.18)	-0.08 (0.17)	0.038
CCT (μm)	15	157 (95)	168 (63)	-3 (40)	0.125

* Difference = After switching – Baseline.

IVBr, intravitreal injection of brolucizumab; Log MAR, logarithm of the minimum angle of resolution; CRT, central subfield retinal thickness; CRV, central retinal volume; eCRV, central retinal volume excluding eyes with subsequent intraocular inflammation; PEDV, subretinal pigment epithelium volume; CCT, central choroidal thickness; PNV, Pachychoroid driven macular neovascularization.

Statistical analysis of CRT, PEDV, and CRV excluded 5 or 6 eyes due to tracing errors. Following the switch to IVBr, significant improvements were observed in CRT (from 247 (130) to 227 (94) μm, P<0.001), PEDV (from 0.18 (0.47) to 0.11 (0.19) mm3, P = 0.04), and Central Choroidal Thickness (CCT) (from 159 (118) to 168 (78) μm, P = 0.018), however, CRV showed no significant change (from 9.80 (1.40) to 9.70 (1.30) mm3, P = 0.111). Here we found that CCT showed a significant paired reduction, although the unpaired group medians were 159 and 168 μm (**Table 1**). In sub-group analysis of PNV and non-PNV eyes, the significant decrease in CRT and PEDV were also observed, while the significant decrease in CCT was observed only in PNV eyes (**Table 1**).

The percentage of eyes without fluid in each retinal compartment tended to increase from pre-IVBr to post-IVBr visits (Intraretinal Fluid (IRF): 61% to 78%, Subretinal Fluid (SRF): 7 to 43%, Subretinal Pigment Epithelial Fluid (SPEF): 4 to 13%) (**Table 2**). Specifically, the percentage of eyes without SRF increased significantly (P = 0.008). A similar trend of increase in the percentage of eyes without fluid in each retinal compartment was observed in PNV and non-PNV eyes (**Table 2**).

**Table 2:** Percentage of eyes without fluid in each retinal compartment at the baseline and after switching to IVBr.

All 23 eyes	Baseline	After switching	P value
IRF, n (%)	14 (61%)	18 (78%)	0.250
SRF, n (%)	2 (7%)	10 (43%)	0.008
SPEF, n (%)	1 (4%)	3 (13%)	0.500
PNV 8 eyes	Baseline	After switching	P value
IRF, n (%)	5 (63%)	7(88%)	1.000
SRF, n (%)	1 (13%)	5 (63%)	0.125
SPEF, n (%)	1 (13%)	3 (38%)	0.500
Non-PNV 15eyes	Baseline	After switching	P value
IRF, n (%)	9 (60%)	11 (73%)	0.500
SRF, n (%)	1 (7%)	5 (33%)	0.125
SPEF, n (%)	0 (0%)	0 (0%)	N/A

IRF, intraretinal fluid; SRF, subretinal fluid; SPEF, sub-pigment epithelium fluid; PNV, Pachychoroid driven macular neovascularization.

**Figure 1:**
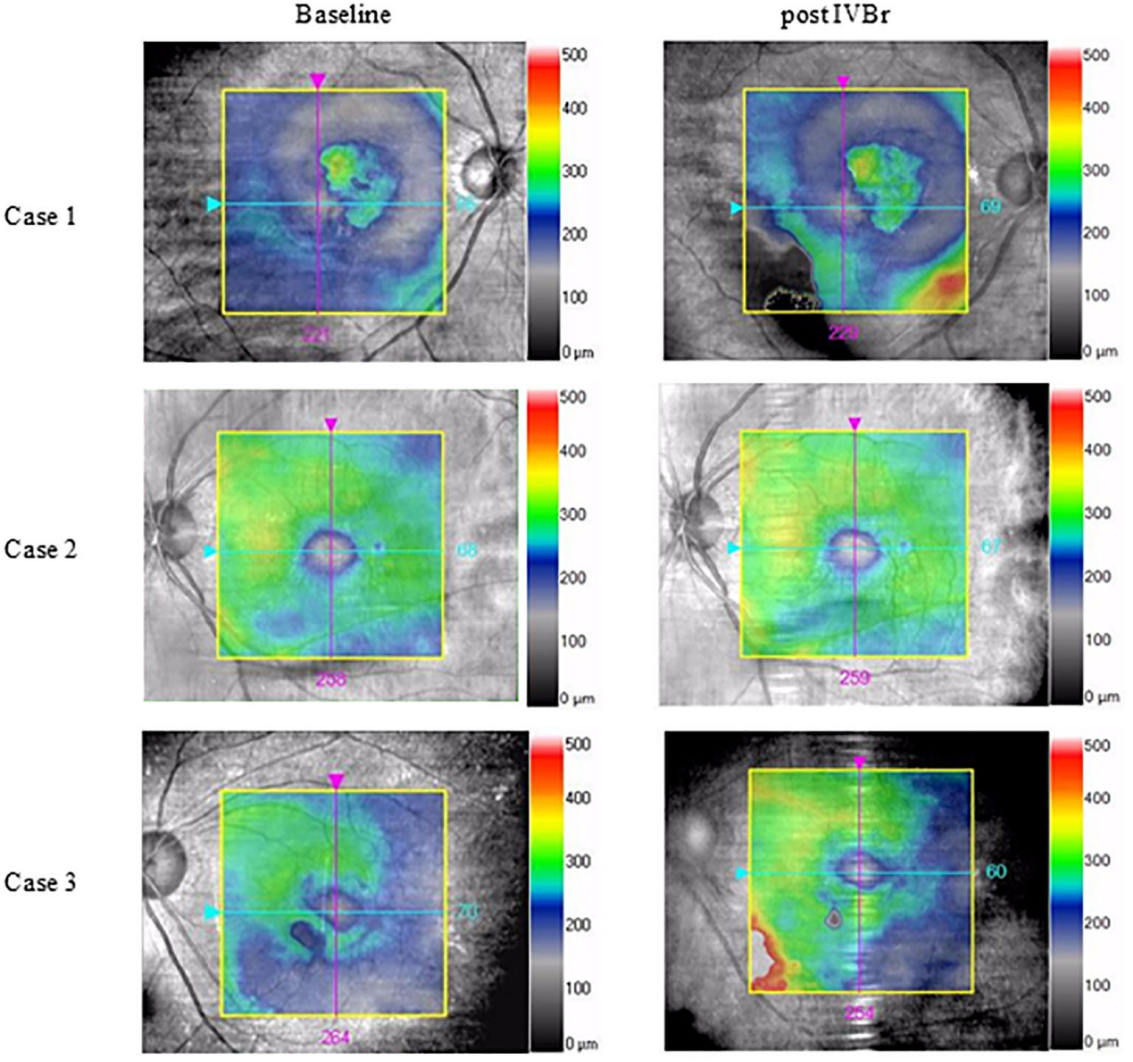
Three cases of intraocular inflammation following IVBr. Color code maps of macular showed retinal thickness in the area of 6 x 6 mm cube scan. Images in the same line were from the identical cases. Images in the left or right row were at the baseline or post-IVBr day 2, respectively. Note that the retinal thickness increased along the arcade vessels following intraocular inflammation.

Three eyes exhibited IOI, observed on post-injection day 2 after the second IVBr. Two eyes exhibited only mild anterior chamber inflammation, while one eye exhibited vasculitis obliterans. Of the two eyes with initial mild anterior chamber inflammation, one developed vasculitis 2 weeks later. In these three eyes, CRV increased, likely due to retinal edema associated with vasculitis, whereas CRT, PEDV and CCT tended to decrease, reflecting the satisfactory efficacy of brolucizumab compared to previous agents at 2 days post injection (**Figure 1**; **Table 2**). When these three eyes were excluded from the analysis, CRV (defined as eCRV) significantly decreased from the pre-IVBr to the post-IVBr visits (9.90 (1.45) to 9.70 (1.20) mm3, P = 0.002, **Table 1**).

## Discussion

Previous studies have demonstrated the short-term efficacy of switch to brolucizumab in patients with nAMD, reporting resolution of retinal fluids such as intraretinal, subretinal, and sub-RPE fluids following the switch (9–13). Enríquez et al. reported significant reduction of central subfield thickness and complete resolution of retinal fluid in 44.2% of eyes with fluid after single injection of brolucizumab (13). In this study, we also observed significant reductions in CRT and retinal and sub-RPE fluids exhibiting a tendency toward rapid resolution after the switch. Additionally, we observed significant reductions in eCRV (CRV excluding three cases with IOI) and PEDV, which were not assessed in previous studies, validated the utility for assessing neovascularization and its exudative distribution in three dimensions.

Although the resolution of retinal fluids in treatment-naïve nAMD is frequently associated with improved visual acuity outcomes (22), our study did not demonstrate a corresponding improvement in visual acuity despite the rapid decrease in retinal fluids and significant reduction in CRT. This observation is consistent with previous reports of switch therapy (9–13), and may indicate long-term deterioration of retinal function prior to the switch. These findings suggest that switch therapy should be promptly considered when previous treatments become ineffective, although it remains uncertain whether visual recovery can be achieved following fluid resolution during long-term follow-up.

In this study, subgroup analysis of PNV and non-PNV-related eyes were performed, although the analysis should be interpreted cautiously due to small number of cohorts. Brolucizumab demonstrated a comparable effect on both PNV and non-PNV-related eyes except for CCT. PNV represents a significant etiology of nAMD in the Japanese population and is characterized by choroidal thickening (17, 23–25). The observed significant reduction in choroidal thickness following the switch to IVBr in PNV-related eyes suggests that brolucizumab may exhibit superior efficacy compared to aflibercept or ranibizumab in targeting choroidal vasculature, as previously reported (26). If choroidal thickening in eyes with PNV serves as a catalyst for neovascularization, then the reduction in choroidal thickness following anti-VEGF therapy could potentially result in PNV regression, rendering PNV a promising target for IVBr.

IOI remains a significant concern with brolucizumab, as retinal vasculitis and vascular occlusion can lead to irreversible declines in visual acuity or loss of visual fields (14, 18, 27, 28). The incidence of IOI has been reported to be higher in the Japanese population (9.4 - 15.4%) than in the North American or European populations (2.4 - 4.6%) (14, 29–31). This study observed a relatively high incidence of IOI 3/23 (13%), consistent with other Japanese reports. However, visual acuity improved following steroid treatment (instillation and/or sub-tenon injection).

In all eyes with IOI, the CRV increased at post-injection day 2, and retinal edema was evident along the macular vasculature in OCT color-coded retinal thickness maps (**Figure 1**). Despite this, CRT consistently decreased after switching to brolucizumab, even in eyes with retinal vasculitis. This discrepancy may be attributed to increased retinal volume along the vasculitis but decreased retinal thickness at the fovea due to the regression of macular neovascularization (**Table 3**). This observation may facilitate the early detection of retinal vasculitis. Further data accumulation is necessary to validate CRV as a potential warning sign of retinal vasculitis in IOI.

**Table 3:** Change of parameters at the baseline and after the second IVBr in eyes with IOI.

Case 1	Baseline	After the second IVBr	Difference *
Log MAR unit	0.70	0.52	-0.18
CRT (μm)	145	72	-73
CRV (mm^3^)	10.1	10.3	+0.2
PEDV (mm^3^)	0.16	0.07	-0.09
CCT (μm)	85	72	-13
Case 2	Baseline	After the second IVBr	Difference *
Log MAR unit	0.52	0.52	0.00
CRT (μm)	132	122	-10
CRV (mm^3^)	8.9	9.7	+0.8
PEDV (mm^3^)	1.12	0.07	-1.05
CCT (μm)	134	123	-11
Case 3	Baseline	After the second IVBr	Difference *
Log MAR unit	1.00	0.52	-0.48
CRT (μm)	165	120	-45
CRV (mm^3^)	7.5	8.4	+0.9
PEDV (mm^3^)	0.06	0.05	-0.01
CCT (μm)	159	160	+1

* Difference = After switching – Baseline.

A negative difference in log MAR unit indicates improvement.

IOI, Intraocular inflammation; Log MAR, logarithm of the minimum angle of resolution; CRT, central subfield retinal thickness; CRV, central retinal volume; PEDV, subretinal pigment epithelium volume; CCT, central choroidal thickness.

This study has several limitations, including its retrospective nature and the small number of patients analyzed. Larger multicenter prospective studies are required to confirm these findings. Additionally, errors in tracing retinal layers by built-in software led to the exclusion of five (22%) cases from CRT and CRV analysis and six (26%) cases from PEDV analysis. This may preferentially retain cases with better imaging quality and more stable disease, limiting generalizability. Future advancements in built-in software utilizing artificial intelligence are essential for improving the accuracy and clinical utility of these parameters. Moreover, the *post hoc* analysis of CRV excluding IOI cases risks confirmation bias and needs further prospective validation.

## Conclusion

This study demonstrated that switching to brolucizumab resulted in rapid decreases in central foveal parameters in Japanese nAMD patients who were resistant to aflibercept or ranibizumab therapy. Notably, a discordant pattern between increasing CRV and improving central foveal parameters may flag incipient brolucizumab-associated IOI and should be validated prospectively.

## Data Availability

The raw data supporting the conclusions of this article will be made available by the authors, without undue reservation.
